# Hybrid lipid-nanogels: nanolipogels for mRNA delivery

**DOI:** 10.1039/d6ra07548g

**Published:** 2026-09-28

**Authors:** Ellen Oudkerk-Sodia, Runxin Xu, Renee Chu, Rupali Dabas, Sofia Patri, Christopher Tidd, Luka Blagojevic, Rüdiger Woscholski, Nazila Kamaly

**Affiliations:** a Department of Chemistry, Imperial College London, Molecular Sciences Research Hub 80 Wood Lane London W12 0BZ UK n.kamaly@imperial.ac.uk r.woscholski@imperial.ac.uk

## Abstract

Nanoscale drug-delivery systems are central to translating mRNA and other biologic medicines into affordable, scalable products. Existing lipid- and polymer-based carriers are often constrained by costly multi-step syntheses, batch variability and acute immune responses driven by ionisable lipids. Here, we report nanolipogels (NLGs), a hybrid phospholipid–polymer carrier built around a synthetic phospholipid, phosphatidylbutenol, synthesised in a single enzyme-catalysed transphosphatidylation step from 1,2-dioleoyl-*sn*-glycero-3-phosphocholine. The reactive vinyl-terminated headgroup permits aqueous, one-pot free-radical co-polymerisation with either the permanently charged cationic comonomer 2-(acryloyloxy)ethyl trimethylammonium chloride (AETC) or the pH-responsive comonomer 2-(dimethylamino)ethyl methacrylate (DMAEMA), facilitating the electrostatic loading of mRNA. The resulting NLGs are sub-200 nm in size, encapsulate green fluorescent protein (GFP) mRNA with efficiencies above 75%, and exhibit sustained mRNA release over seven days in the presence of 10 mM glutathione. In HeLa and brain endothelial bEnd.3 cells, NLGs showed cell viability comparable to optimised liposomal controls and substantially higher than that observed with Lipofectamine 2000, while DMAEMA-NLGs produced a ∼10.9-fold increase in GFP fluorescence over Lipofectamine 2000. The combination of benign, scalable chemistry with efficient mRNA delivery positions NLGs as a promising platform for accessible nanomedicine.

## Abstract 2

Best of both worlds: nanolipogels deliver mRNA to cells post cellular entry and endosomal release and outperform standard transfection reagents.

## Graphical abstract

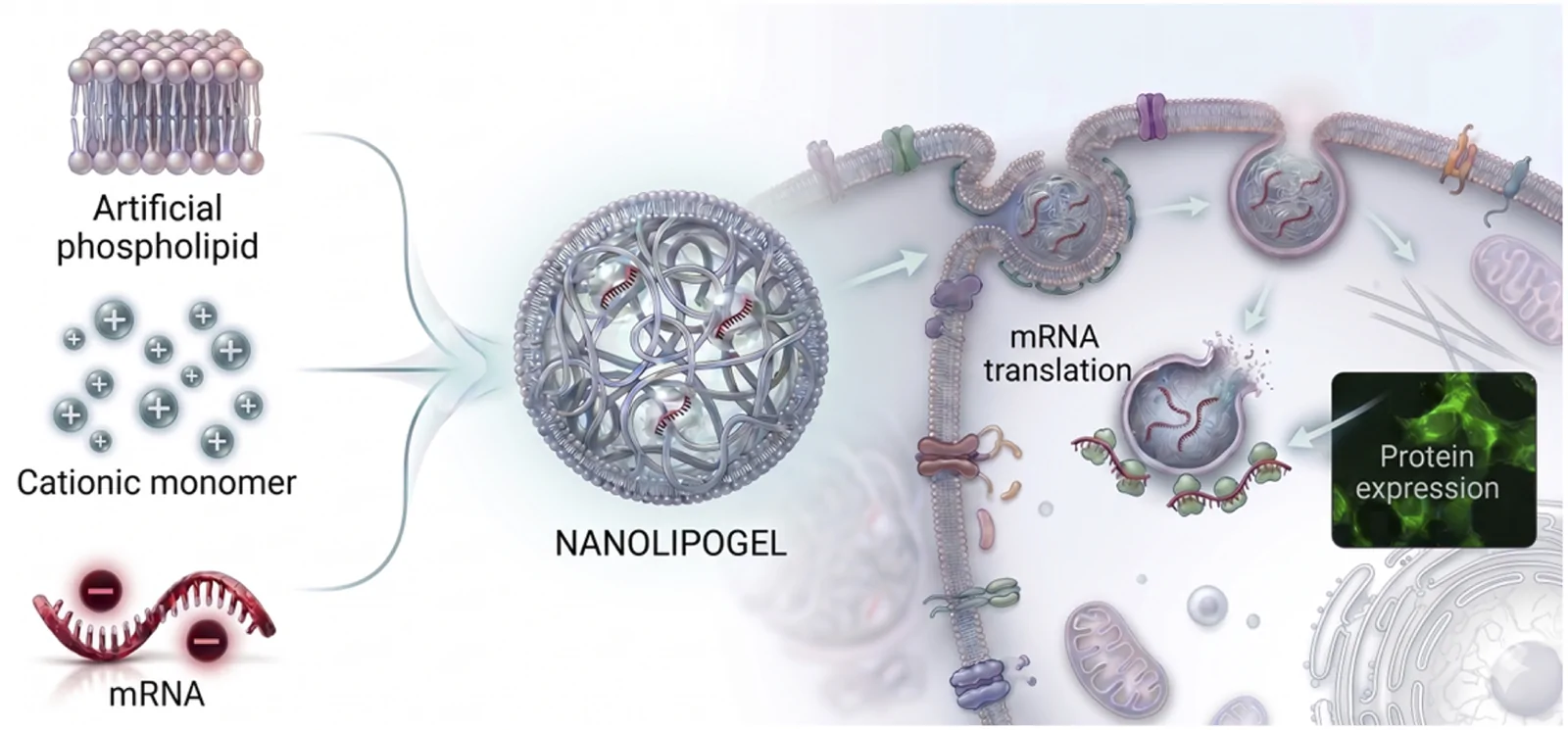

## 1 Introduction

The most promising biologic medicines, including messenger RNA (mRNA), small interfering RNA and protein therapeutics, require sophisticated delivery systems to cross biological barriers, evade nuclease degradation and reach the cells of interest.^1,2^ Nanoparticles (NPs) provide a tunable chemical platform to address these constraints: their size, surface chemistry and composition can be engineered to traverse biological barriers, respond to physiological triggers such as pH or redox gradients, and deliver labile cargoes selectively to diseased tissue while sparing healthy cells.^3–5^ Among the cargoes amenable to nanoparticle delivery, mRNA stands out for its ability to direct the *in situ* synthesis of therapeutic proteins and antigens with high target specificity.^6–8^ Lipid-based nanocarriers, including liposomes, micelles and lipid nanoparticles (LNPs), have been among the most successful clinical platforms for nucleic-acid delivery to date. Their constituents are largely biocompatible and can accommodate sizeable polynucleotide cargoes, but they are also vulnerable to enzymatic hydrolysis and disruption by hydrophobic environments, and the ionisable lipids that drive endosomal escape can elicit acute innate-immune responses and longer-term toxicity.^10–13^ Polymeric nanogels are nanoscale, cross-linked polymer networks and offer complementary mechanical robustness, high water content and tunable softness, properties that translate into prolonged circulation, sustained release and reduced off-target toxicity.^14–16^

In this work, we report nanolipogels (NLGs): hybrid lipid–polymeric nanocarriers built around a polymerisable synthetic phospholipid, phosphatidylbutenol. Phosphatidylbutenol is obtained in a single step from 1,2-dioleoyl-*sn*-glycero-3-phosphocholine (DOPC) by an enzyme-catalysed transphosphatidylation, in which phospholipase D (PLD) from *Streptomyces* sp. exchanges the choline headgroup of DOPC for a vinyl-terminated 3-buten-1-ol moiety (Scheme 1).^17^ Enzymatic synthesis preserves the acyl tails and avoids the harsh conditions of conventional phospholipid chemistry, and is amenable to scale-up under mild aqueous conditions, which are both important features for accessible manufacture. The terminal alkene of phosphatidylbutenol then serves as a polymerisable handle in a one-pot, room-temperature aqueous free-radical polymerisation initiated by ammonium persulfate (APS) and *N*,*N*,*N*′,*N*′-tetramethylethylenediamine (TEMED), avoiding the volatile organic solvents and high temperatures of nanoprecipitation or inverse-emulsion approaches.^18–20^

**Scheme 1:**
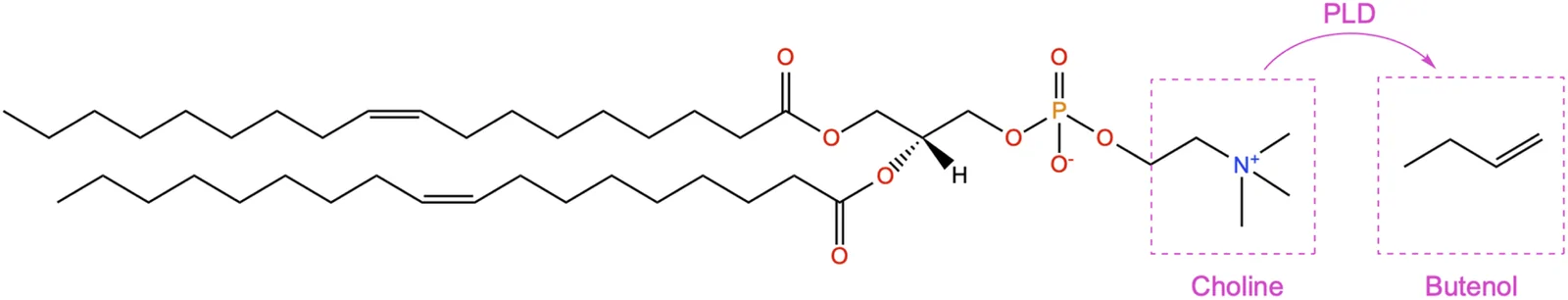
Enzyme-catalysed transphosphatidylation of DOPC. Phospholipase D (PLD) from *Streptomyces* sp. exchanges the choline headgroup for a 3-buten-1-ol moiety to yield the polymerisable artificial phospholipid phosphatidylbutenol.

Polymerisable phospholipids have a long history in membrane science. Lipids bearing diacetylene, acryloyl, methacryloyl or sorbyl functionality, introduced either in the acyl tails or at the headgroup, have been used to convert self-assembled bilayers into covalently stabilised polymerised liposomes with reduced permeability and improved resistance to detergents and serum.^32,33^ These systems have generally required multi-step total synthesis of the lipid monomer, which constrains both scale and cost. A complementary strategy is the lipid–polymer hybrid nanoparticle, in which a preformed polymer core is coated with a physically adsorbed lipid monolayer, combining the colloidal stability of the polymer with a biocompatible lipid surface. In these architectures, the two components remain non-covalently associated and can dissociate on dilution or on exposure to serum proteins.^34^ The NLGs reported here differ from both classes. The lipid is itself the network-forming monomer, so the lipid and polymer phases are covalently connected rather than physically assembled, and the polymerisable handle is installed enzymatically in a single step from a commodity phospholipid rather than by *de novo* synthesis.

To electrostatically capture the polyanionic mRNA cargo, phosphatidylbutenol is co-polymerised with one of two cationic comonomers. The quaternary acrylate 2-(acryloyloxy)ethyl trimethylammonium chloride (AETC) presents a permanent positive charge that has been used in nucleic-acid-binding scaffolds,^21,22^ but high cationic-charge densities can drive oxidative stress and immunogenicity.^23^ 2-(Dimethylamino)ethyl methacrylate (DMAEMA), in contrast, is a pH-responsive tertiary-amine methacrylate whose protonation state varies with physiological pH and whose polyplexes have shown promise in gene delivery.^9,24–27^ We compare the two systems for mRNA encapsulation, controlled release and transfection efficiency in HeLa cells, and evaluate biocompatibility in the brain microvascular endothelial line bEnd.3 as a model of sensitive vascular tissue. Cationic monomers of this type are well established in nucleic-acid delivery: poly(DMAEMA) and its copolymers were among the earliest synthetic transfection polymers, the tertiary amine (p*K*_a_ ≈ 7.4) conferring partial protonation at physiological pH and additional buffering capacity in the acidifying endosome, whereas quaternary ammonium acrylates such as AETC carry a pH-independent permanent charge that binds nucleic acids more tightly but provides no buffering.^25^ The advance reported here lies in three specific elements: (i) the single-step, enzyme-catalysed conversion of a commodity phospholipid into a polymerisable lipid monomer; (ii) an aqueous, solvent-free, room-temperature co-polymerisation that encapsulates cargo *in situ* during network formation rather than by post-loading; and (iii) the resulting covalently crosslinked lipid–polymer network, in which the comparison between a permanently charged and a titratable comonomer provides an internal test of the proposed pH-dependent release mechanism.

## 2 Experimental

### 2.1 Materials

1,2-Dioleoyl-*sn*-glycero-3-phosphocholine (DOPC) and 1,2-dioleoyl-*sn*-glycero-3-phospho-l-serine (DOPS) were purchased from Avanti Polar Lipids. Phospholipase D (PLD) from *Streptomyces* sp. (Type VII), 3-buten-1-ol, 2-(dimethylamino)ethyl methacrylate (DMAEMA), 2-(acryloyloxy)ethyl trimethylammonium chloride (AETC, 80 wt% in H_2_O), *N*,*N*,*N*′,*N*′-tetramethylethylenediamine (TEMED), ammonium persulfate (APS), bovine serum albumin (used solely as an enzyme stabiliser in the transphosphatidylation reaction), Nile Blue acrylamide, cholesterol, glutathione (GSH), uranyl acetate, Hoechst 33342, dimethyl sulfoxide (DMSO), formaldehyde solution (4 wt%) and Triton X-100 were purchased from Merck/Sigma-Aldrich. CleanCap® EGFP mRNA (5moU) was obtained from TriLink Biotechnologies. Lipofectamine™ 2000, cell-culture media and solvents were purchased from Thermo Fisher Scientific. Cell Counting Kit-8 (CCK-8) was supplied by Dojindo. The Quant-iT™ RNA, mMESSAGE mMACHINE™ T7, Poly(A) Tailing and RNeasy® Mini kits were used according to the manufacturers' instructions. HeLa cells (ATCC CCL-2) and bEnd.3 cells (ATCC CRL-2299) were purchased from ATCC. DEPC-treated, nuclease-free water (Fisher BioReagents) was used for all mRNA work. All other chemicals were of analytical grade and used without further purification.

### 2.2 Synthesis of phosphatidylbutenol

A 5 mL round-bottomed flask was charged with an aqueous phase (4.5 mL; 200 mM 3-buten-1-ol, 0.05 mg mL^−1^ bovine serum albumin, 0.045 U PLD per 100 µL, 100 mM sodium acetate buffer, pH 5.6) and an organic phase (4.5 mL; 2 mM DOPC in chloroform). The biphasic mixture was stirred at 250 rpm and 41 °C for 2 h, then centrifuged at 13 000 rpm for 3 min. The organic phase containing phosphatidylbutenol was collected and stored at −20 °C. Batches of insufficient purity were polished by silica-gel column chromatography (Geduran® Silicagel 60, CHCl_3_/MeOH 19 : 1 → 9 : 1). The product was routinely obtained at ≥95% purity as a colourless oil.

^1^H NMR (400 MHz, CDCl_3_) *δ* 5.78 (q, 1H), 5.34 (qd, 4H), 5.24 (q, 1H), 5.10 (ddt, 1H), 5.01 (ddt, 1H), 4.40 (m, 2H), 4.21–4.14 (m, 2H), 3.93 (t, 2H), 2.37–2.20 (m, 6H), 2.01 (q, 8H), 1.58 (*p*, 4H), 1.29 (m, 28H), 0.88 (t, 6H). ^13^C NMR (101 MHz, CDCl_3_) *δ* 173.8, 134.7, 130.2, 117.0, 70.9, 70.7, 65.3, 63.0, 34.5, 34.3, 32.1, 29.9, 29.7, 29.5, 29.4, 29.4, 27.4, 25.1, 22.8, 14.3. ^31^P NMR (162 MHz, CDCl_3_) *δ* −1.05. FT-MS (C_43_H_77_O_8_P^−^): calcd 753.5440, found 753.5435. MALDI-ToF (C_43_H_79_O_8_P): [M + Na]^+^ calcd 777.07, found 777.52. FT-IR and MALDI-ToF spectra are provided in Fig. S1–S4.

### 2.3 Synthesis of empty NLGs

Milli-Q water was sparged with N_2_ for ≥40 min. Phosphatidylbutenol (0.5 mg) in chloroform was dried under N_2_, redispersed in 1 mL of deoxygenated water with sonication (9 min) and stirred under N_2_ for 30 min. APS (1 mg in 1 mL water) was added, followed by TEMED (1.5 µL in 0.5 mL water), and polymerisation was carried out under N_2_ for 1 h at room temperature. NLGs were purified using 100 kDa Amicon® Ultra-15 filters at 1200*g* with three water washes and stored at 4 or −20 °C. Control particles for the polymerisation-dependence experiment were prepared identically but with the APS initiator omitted.

### 2.4 *In vitro* transcription of GFP mRNA

GFP mRNA was transcribed *in vitro* from pEGFP (plasmid #76015) bearing a T7 promoter using the Invitrogen mMESSAGE mMACHINE T7 kit. The product was polyadenylated (Invitrogen Poly(A) Tailing kit) and purified (RNeasy Mini kit). Concentration and purity were determined on a NanoDrop One. The 755-nucleotide GFP mRNA (≈250 kDa) was stored in batches at −80 °C.

### 2.5 Synthesis of mRNA-loaded NLGs

DEPC-treated, nuclease-free water (pH 7.2) was sparged with N_2_ for ≥40 min. AETC (2 µL) or DMAEMA (1.86 µL) was combined with GFP mRNA (6 µg) in 1 mL of deoxygenated water and stirred on ice under N_2_ for 30 min. In parallel, phosphatidylbutenol (0.5 mg) was sonicated into 1 mL of deoxygenated water and combined with the mRNA/comonomer solution. APS (1 mg in 1 mL water) and TEMED (1.5 µL in 0.5 mL water) were added, and polymerisation was carried out on ice under N_2_ for 1 h. NLGs were purified through 100 kDa Amicon Ultra-15 filters and stored at 4 or −20 °C. Cholesterol-modified NLGs were prepared by adding cholesterol together with phosphatidylbutenol.

### 2.6 Synthesis of fluorescently labelled NLGs

Nile-Blue-labelled NLGs were synthesised exactly as described in Section 2.5, substituting 5 mol% of the cationic comonomer for Nile Blue acrylamide (Fig. S11). The terminal acrylamide of the dye is incorporated into the growing free-radical chain so that the dye is covalently bound, eliminating leaching during incubation with cells. The resulting Nile-Blue-NLGs retained a sub-200 nm hydrodynamic diameter and >70% mRNA encapsulation efficiency.

### 2.7 Preparation of control liposomes

Cholesterol/DOPC/DOPS (5 : 4 : 1, 30 µmol total lipid)^28^ was dried under N_2_, hydrated in 10 mL of deionised water and extruded 11 times at 60 °C through a 200 nm polycarbonate membrane. Liposomes were stored at 4 °C.

### 2.8 Physicochemical characterisation

Hydrodynamic diameter, polydispersity index (PDI) and zeta potential were measured on a Malvern Zetasizer at 25 °C in filtered Milli-Q water (*n* = 3, 25 sub-runs each). For TEM, 3.5 µL of NLG suspension was deposited on a glow-discharged 300-mesh lacey-carbon copper grid for 1 min, blotted, stained with 3.5 µL of 2% w/w uranyl acetate for 1 min, blotted again and imaged on a Talos F200X G2 microscope at 450 00×. To test the contribution of polymerisation to particle integrity, NLGs prepared with and without APS were challenged with methanol and chloroform and examined by XPS. Full details of the NMR, FT-IR, XPS, MALDI-ToF, plate-reader, confocal and live-cell imaging instrumentation are provided in Section S1 of the SI.

### 2.9 Encapsulation efficiency

mRNA encapsulation was determined indirectly by quantifying free mRNA in the filtrate after centrifugal ultrafiltration (Amicon Ultra 100 kDa MWCO, 4 °C, 14 000*g*, 15 min) using the Quant-iT RiboGreen assay against the calibration curve in Fig. S5, with confirmation on a NanoDrop One. Encapsulation efficiency (% EE) was calculated as:

1 % EE = (1 − [free mRNA]/[total mRNA]) × 100

### 2.10 mRNA release studies

mRNA-loaded NLGs were incubated with 10 mM GSH (pH 3.3) at 37 °C with stirring (100 rpm). At defined time points the dispersion was filtered through a 100 kDa Amicon Ultra-0.5 device, mRNA in the filtrate was quantified on a NanoDrop One, and the retentate was reconstituted in fresh release medium.

### 2.11 Cell culture and cytotoxicity

HeLa and bEnd.3 cells were cultured in DMEM supplemented with 10% FBS and 1% penicillin–streptomycin at 37 °C and 5% CO_2_; all experiments used cells in logarithmic growth. For viability assays, cells were seeded at 5000 per well in 96-well plates (100 µL medium) and allowed to attach for 24 h. Medium was replaced with NLGs (0–250 µg mL^−1^ in PBS, pH 7.3), control liposomes or Lipofectamine 2000 for a further 24 h, and viability was measured using the CCK-8 assay according to the manufacturer's protocol.

### 2.12 Confocal-microscopy uptake

HeLa cells were seeded at 25 000 per well in µ-Slide 8-well chambers (Ibidi) and incubated for 24 h. Cells were then exposed to Nile-Blue-labelled mRNA-NLGs in PBS, washed with PBS, fixed with 4% paraformaldehyde (10 min), washed, stained with Hoechst 33342 (100 ng mL^−1^, 15 min) and imaged on a Leica TCS SP8 DLS inverted microscope using LAS X. Images were processed in Fiji.

### 2.13 Transfection

HeLa cells were seeded at 10 000 per well in 96-well plates (100 µL medium) and allowed to attach for 24 h. Cells were then treated with 50 µg mL^−1^ mRNA-NLGs in OptiMEM (pH 8.4), or with Lipofectamine 2000 according to the manufacturer's protocol. Normalised GFP fluorescence was acquired 24 h post-treatment by IncuCyte live-cell imaging and quantified as total green-object integrated intensity (GCU × µm^2^ per image). The transfection experiment was performed as a proof-of-concept study with one well per condition.

### 2.14 Statistical analysis

Quantitative data are presented as mean ± standard deviation (s.d.) of at least three independent biological or synthetic replicates, unless otherwise stated. Pairwise comparisons were tested by two-tailed Student's *t*-test, and multi-group comparisons across NLG concentration in the cell-viability assay were tested by one-way ANOVA followed by Tukey's *post-hoc* honest-significant-difference test; *p* < 0.05 was considered statistically significant. Transfection quantification was performed as a proof-of-concept study with one well per condition and was therefore evaluated descriptively without inferential statistical testing.

## 3 Results and discussion

### 3.1 Synthesis of phosphatidylbutenol

Phosphatidylbutenol was prepared by a two-phase, PLD-catalysed transphosphatidylation of DOPC with 3-buten-1-ol and routinely obtained at ≥95% purity. Successful synthesis was confirmed by ^1^H, ^13^C and ^31^P NMR, FT-MS, FT-IR and MALDI-ToF mass spectrometry (Section 2.2); the FT-IR C=C stretch and the MALDI-ToF [M + Na]^+^ ion confirmed the terminal alkene (Fig. S1–S4).

### 3.2 Synthesis and characterisation of empty NLGs

Phosphatidylbutenol was self-assembled in deoxygenated water and polymerised in one pot with APS and TEMED at room temperature. Empty NLGs were reproducibly obtained with an average hydrodynamic diameter of 136 nm, PDI 0.30 and a strongly negative zeta potential (−69.8 mV) consistent with exposure of the anionic phosphate headgroup at the particle surface. Spherical sub-200 nm morphology was confirmed by transmission electron microscopy (TEM). To confirm that polymerisation, rather than simple lipid self-assembly, was responsible for particle integrity, NLGs were prepared with and without the APS initiator and challenged with methanol and chloroform. XPS showed that APS-polymerised NLGs lost the headgroup C=C signature, consistent with consumption of the polymerisable alkene, and these particles resisted methanol while APS-free particles dissolved (Fig. S3). Both populations dissolved in chloroform, indicating that lipid hydrophobic interactions also contribute to integrity and that an additional cross-linker would further stabilise the network. These observations indicate that the alkene is consumed and that the network is stabilised, but do not by themselves quantify incorporation of phosphatidylbutenol into the cationic copolymer.

### 3.3 Synthesis and characterisation of mRNA-loaded NLGs

For mRNA delivery, the cationic comonomer AETC or DMAEMA was incorporated to provide electrostatic binding sites for green-fluorescent-protein (GFP)-encoding mRNA (Scheme 2). Empty AETC- and DMAEMA-NLGs were both sub-200 nm; the slightly smaller, less-negative AETC-NLGs are consistent with the permanent positive charge of AETC screening the anionic phospholipid surface more efficiently (Fig. S6).

**Scheme 2:**
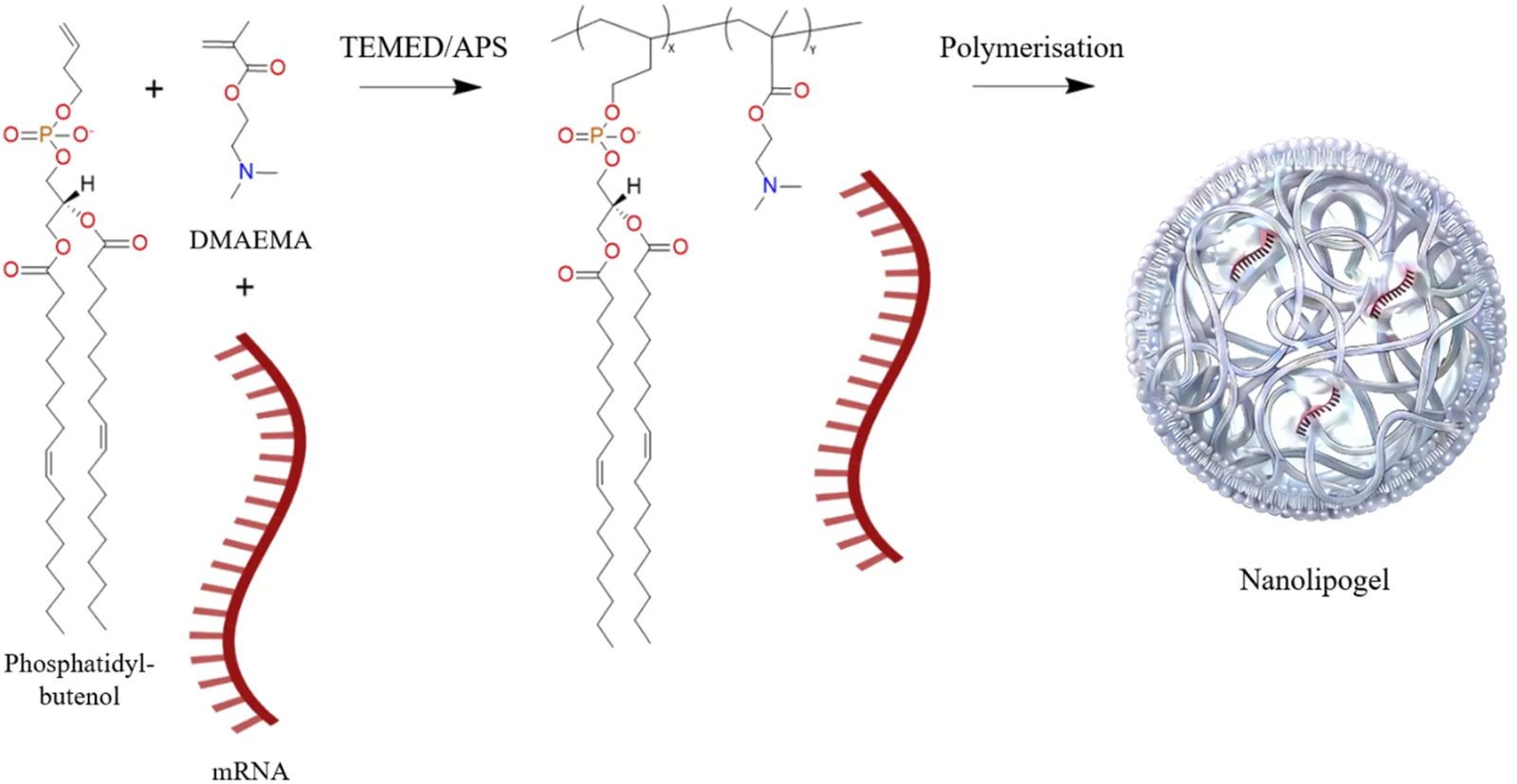
Synthesis of mRNA-loaded NLGs by aqueous one-pot free-radical co-polymerisation of phosphatidylbutenol with the cationic comonomer DMAEMA (or AETC). Cationic monomers condense mRNA into circular subdomains within the lipid–polymer network. The relative sizes of the components are depicted schematically and are not to scale.

Both mRNA-AETC-NLGs and mRNA-DMAEMA-NLGs were sub-200 nm with average % EE of 79.2% and 76.8%, respectively (Fig. 1; full DLS data in Fig. S6 and S7). These efficiencies lie within the range reported for cationic polymer- and lipid-based mRNA carriers, although encapsulation efficiency is determined here indirectly, against a RiboGreen calibration (Fig. S5), and is not strictly comparable with values obtained by other separation and quantification methods.^28^ Reactions were carried out in DEPC-treated, nuclease-free water (pH 7.2) on ice; the latter was important for keeping particle size below 200 nm (Fig. S7).

**Fig. 1:**
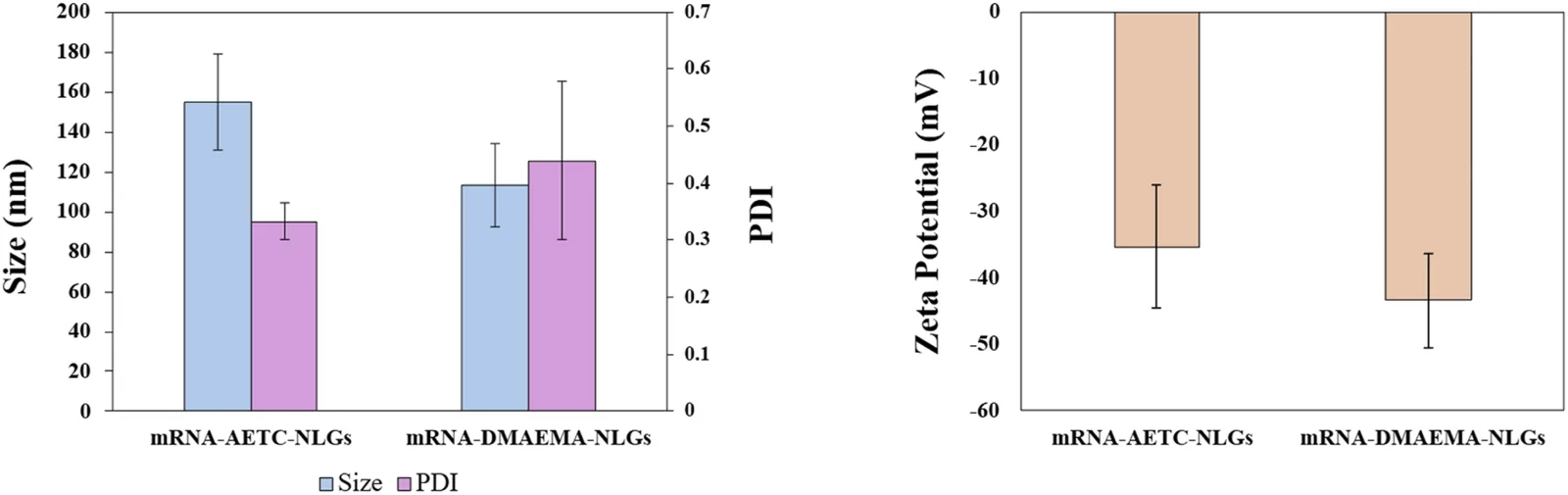
Hydrodynamic diameter (nm), polydispersity index (PDI) and zeta potential (mV) of mRNA-AETC-NLGs and mRNA-DMAEMA-NLGs measured by dynamic light scattering. Data are mean ± s.d., *n* = 3.

### 3.4 Morphology and shelf-life of mRNA-loaded NLGs

Negative-stain TEM confirmed roughly spherical morphology and consistent particle sizes for both formulations (Fig. 2a and b; further micrographs in Fig. S9 and S10). Both populations exhibited internal circular subdomains reminiscent of those reported for LNPs and liposomes,^29,30^ which we tentatively assign to clusters of cationic monomers that condense mRNA. mRNA-DMAEMA-NLGs displayed fewer such subdomains than mRNA-AETC-NLGs, possibly reflecting tighter pH-responsive complexation of mRNA with DMAEMA. Inclusion of cholesterol in mRNA-AETC-NLGs (Fig. 2c) gave more clustered subdomains, consistent with the known role of cholesterol in modulating lipid fluidity and nanodomain formation.^31^ mRNA-DMAEMA-NLGs stored at −20 °C retained both their hydrodynamic diameter and mRNA encapsulation for at least one month (Fig. S8).

**Fig. 2:**
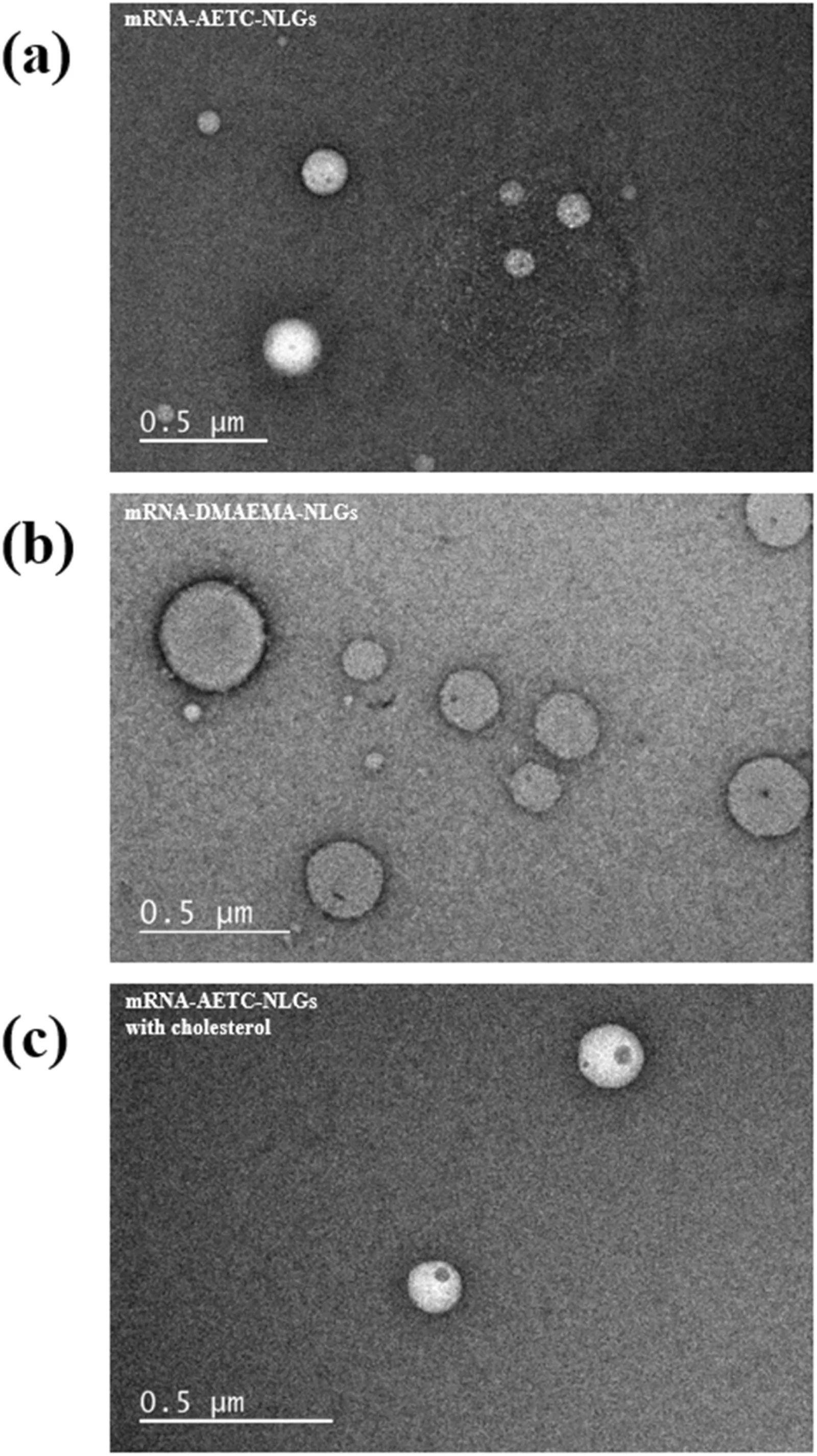
Negative-stain TEM micrographs of (a) mRNA-AETC-NLGs, (b) mRNA-DMAEMA-NLGs, and (c) mRNA-AETC-NLGs incorporating cholesterol.

### 3.5 mRNA release from NLGs

mRNA-AETC- and mRNA-DMAEMA-NLGs were incubated with 10 mM glutathione (GSH) at pH 3.3 and 37 °C (Fig. 3). We note explicitly that the NLG network contains no disulfide or other redox-cleavable linkage, so glutathione is not expected to act as a chemical trigger, and that pH 3.3 lies below the pH of any compartment on the endolysosomal route. Release was therefore measured under a single set of conditions, and the data below establish the relative retentiveness of the two formulations rather than a pH- or redox-triggered release mechanism; a matched panel across physiologically relevant pH values with and without GSH would be required to demonstrate pH-dependence directly. mRNA-AETC-NLGs released 39% within 6 h and 87% over 168 h, while the DMAEMA system was substantially more retentive (24% at 6 h, 67% at 168 h), consistent with stronger pH-modulated complexation between DMAEMA and mRNA. Under these conditions the DMAEMA system is therefore the more retentive of the two, which may be advantageous for prolonged protein expression. Because released mRNA was quantified in the filtrate of a 100 kDa MWCO device while the GFP mRNA used here is 755 nucleotides (≈250 kDa), the absolute release percentages depend on the recovery of free mRNA through this membrane, which was not independently determined here. The values are therefore interpreted as a relative comparison between the two formulations rather than as absolute released fractions.

**Fig. 3:**
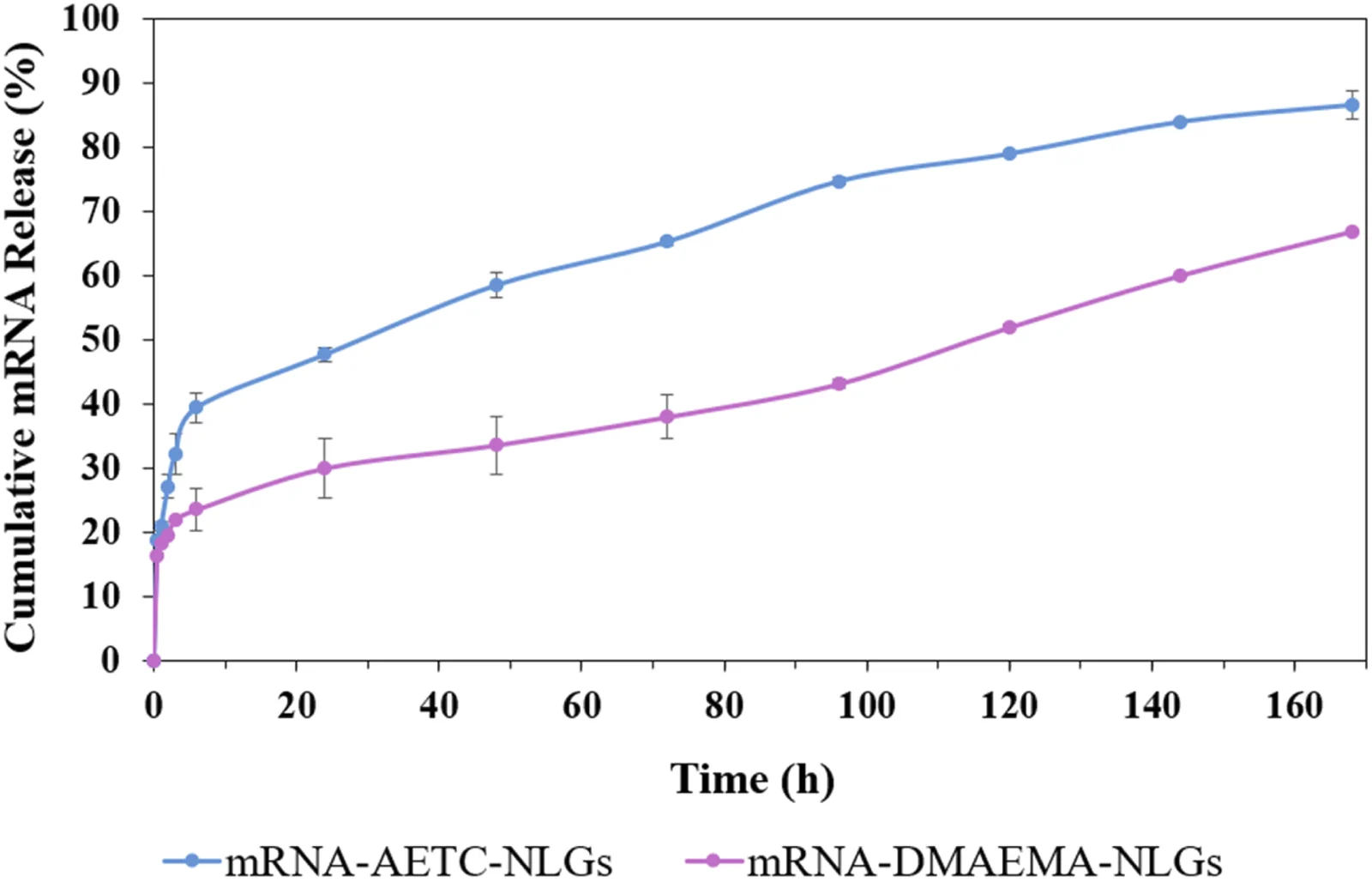
Cumulative mRNA release from mRNA-AETC-NLGs (blue) and mRNA-DMAEMA-NLGs (rose) incubated with 10 mM GSH (pH 3.3) at 37 °C over 168 h. Data are mean ± s.d., *n* = 3.

### 3.6 Cytotoxicity, uptake and transfection

Cytotoxicity of mRNA-NLGs was assessed in HeLa and bEnd.3 cells using the CCK-8 assay, with Lipofectamine 2000 and an optimised cholesterol/DOPC/DOPS liposomal control (5 : 4 : 1 mol%)^28^ as benchmarks. Both NLG formulations supported cell viability at or slightly above 100% up to 250 µg mL^−1^ in HeLa cells, comparable to the optimised liposomes and substantially better than Lipofectamine 2000 (Fig. 4a). The advantage was even more pronounced in bEnd.3 brain endothelial cells, where Lipofectamine 2000 reduced viability to 40% while NLGs preserved viability above 90% (Fig. 4b).

**Fig. 4:**
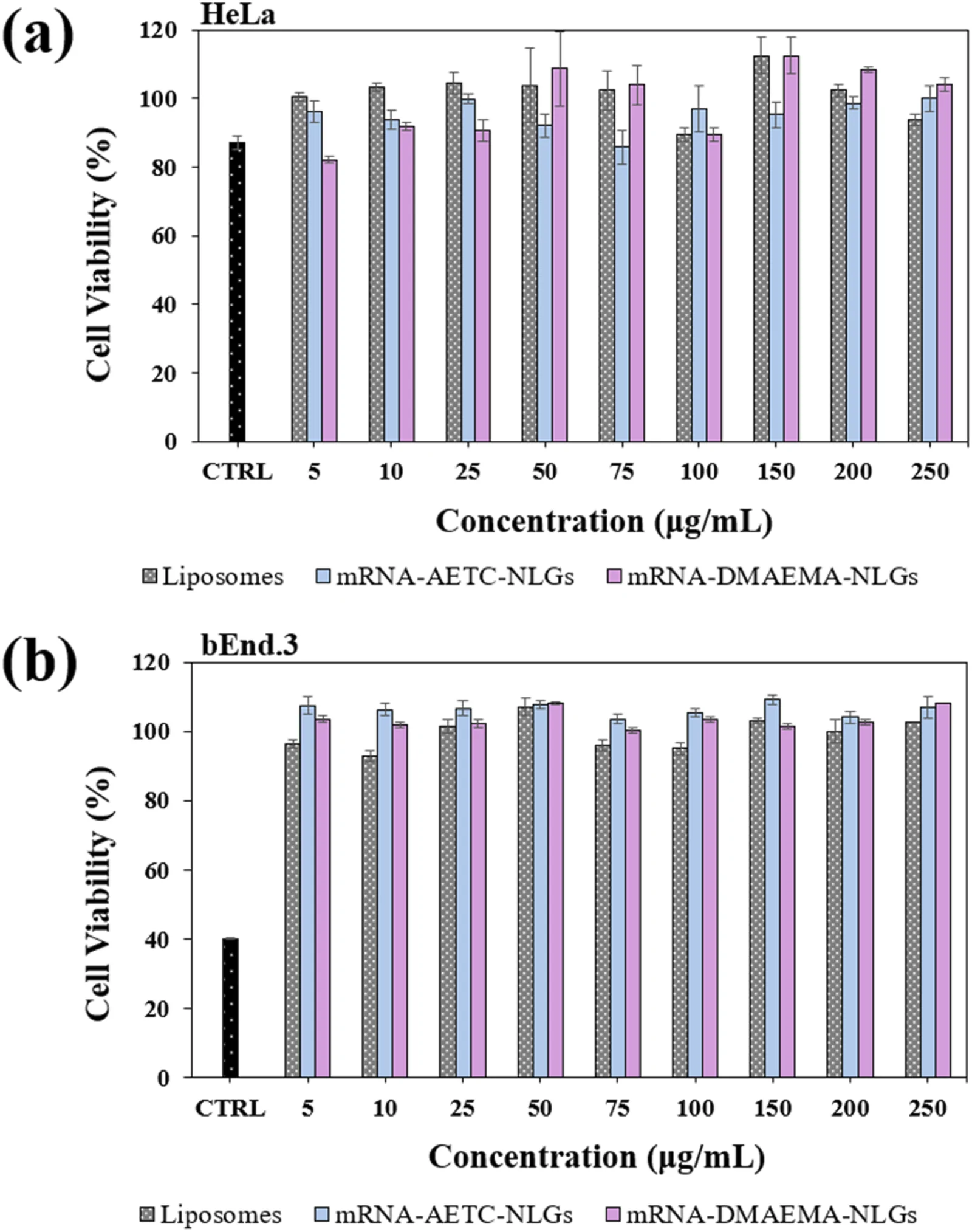
CCK-8 cell-viability assays following 24 h incubation at 37 °C with control liposomes, mRNA-AETC-NLGs and mRNA-DMAEMA-NLGs in (a) HeLa and (b) bEnd.3 cells. CTRL denotes Lipofectamine 2000, applied according to the manufacturer's protocol. Data are mean ± s.d. of three replicates.

For confocal-microscopy uptake studies, NLGs were rendered fluorescent by co-polymerising Nile-Blue acrylamide into the network (Section 2.6 and Fig. S11). The labelled mRNA-AETC-NLGs (170.5 nm; PDI 0.47; −40.9 mV) and mRNA-DMAEMA-NLGs (169.7 nm; PDI 0.35; −30.3 mV) retained the size and surface charge of their unlabelled counterparts. Qualitative confocal-microscopy analysis confirmed intracellular fluorescence in HeLa cells after 24 h at 50 µg mL^−1^ (Fig. 5A), providing visual evidence of cellular uptake despite the net-negative surface charge of these lipid-rich particles.

**Fig. 5:**
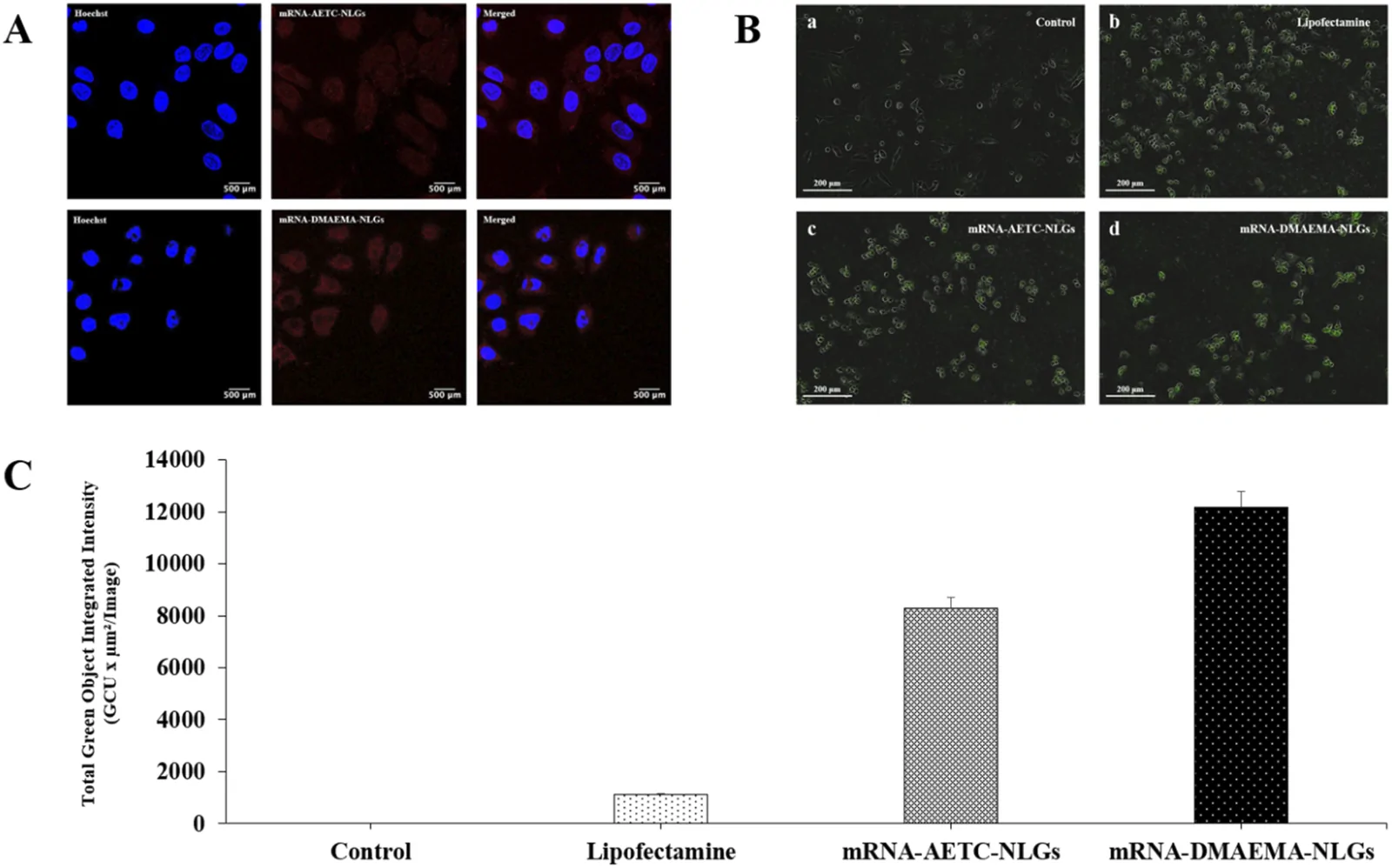
(A) Confocal images of HeLa cells incubated with Nile-Blue-labelled mRNA-AETC-NLGs and mRNA-DMAEMA-NLGs (red, NLGs; blue, Hoechst-stained nuclei). (B) IncuCyte live-cell imaging and (C) quantification of GFP transfection in HeLa cells treated with untreated control, Lipofectamine 2000, mRNA-AETC-NLGs and mRNA-DMAEMA-NLGs (50 µg mL^−1^, 24 h).

GFP expression was subsequently quantified in HeLa cells by IncuCyte live-cell imaging 24 h after exposure to 50 µg mL^−1^ of each formulation (Fig. 5B). Both NLG formulations produced greater normalised GFP fluorescence than Lipofectamine 2000: mRNA-DMAEMA-NLGs showed a ∼10.9-fold increase, and mRNA-AETC-NLGs a ∼7.4-fold increase, in normalised GFP fluorescence (Fig. 5C). As this proof-of-concept experiment was performed with one well per condition, these differences are reported descriptively.

The greater GFP expression observed with the DMAEMA system is consistent with its tighter, pH-responsive condensation of mRNA observed in TEM and release studies, and with its potential for endosomal buffering at acidic pH. This interpretation remains a hypothesis: no colocalisation with endosomal or lysosomal markers, and no assessment of endocytic route or endosomal escape, has been carried out here. GFP expression was also quantified as integrated green-object intensity by live-cell imaging rather than as the percentage of transfected cells by flow cytometry, which limits direct comparison with transfection efficiencies conventionally reported for lipid nanoparticles.

## 4 Conclusions

We have introduced NLGs as a hybrid lipid–polymer platform for mRNA delivery, built on the enzyme-catalysed synthesis of a polymerisable artificial phospholipid, phosphatidylbutenol, and an aqueous one-pot free-radical co-polymerisation with cationic comonomers. The chemistry is mild, scalable and free of organic solvents and high temperatures, directly relevant to manufacturing in low-resource settings. The sub-200 nm particles encapsulate GFP mRNA above 75%, release it slowly under acidic conditions, and outperform Lipofectamine 2000 in HeLa transfection (up to ∼10-fold for the DMAEMA system) while preserving brain-endothelial cell viability. The DMAEMA system in particular combines high transfection efficiency with stronger pH-modulated retention, making it the more promising candidate for further development.

Lipofectamine 2000 is a standard *in vitro* transfection reagent but is not representative of the ionisable-lipid nanoparticles used clinically, which typically comprise an ionisable amino lipid such as SM-102, ALC-0315 or DLin-MC3-DMA together with a helper phospholipid, cholesterol and a PEG-lipid, and which achieve endosomal escape through pH-dependent protonation of the ionisable lipid.^11^ Reported transfection efficiencies for such systems in HeLa cells span a wide range and depend strongly on mRNA construct, dose, serum conditions, incubation time and readout modality, so literature values cannot be compared quantitatively with the data reported here.^29,30^ A matched head-to-head comparison against a benchmark ionisable-lipid nanoparticle formulated and assayed under identical conditions is the necessary next step. Future work will incorporate bifunctional cross-linkers, optimise the comonomer-to-lipid ratio, broaden the cargo to other nucleic-acid modalities and conjugate targeting ligands. The principal advance is conceptual: NLGs embed the cargo-loading and biomimicry of lipid carriers within a covalent polymer network built from a sustainable, enzyme-derived monomer, providing a chemistry that is both performance-competitive and aligned with the global-health imperative of accessible nanomedicine.

## Author contributions

E. O.-S.: conceptualisation, investigation, methodology, formal analysis, visualisation, writing – original draft. R. X., R. C., R. D., S. P., C. T. and L. B.: investigation, methodology, formal analysis, writing – review and editing. R. W. and N. K.: conceptualisation, supervision, project administration, funding acquisition, writing – review and editing.

## Conflicts of interest

There are no conflicts to declare.

## Supplementary Material

RA-OLF-D6RA07548G-s001

## Data Availability

The data supporting this article have been included as part of the manuscript and the supplementary information (SI). Additional raw data are available from the corresponding authors upon reasonable request. Supplementary information: FT-IR, XPS and MALDI-ToF characterisation of phosphatidylbutenol; RiboGreen calibration curve; formulation optimisation and shelf-life data; supplementary electron micrographs; the fluorescent-NLG co-polymerisation scheme; and a statistical summary. See DOI: https://doi.org/10.1039/d6ra07548g.
