# Supplementary material for: Hybrid lipid-nanogels: nanolipogels for mRNA delivery
Source: RSC Adv. 2026 Sep 28;16(46):59320–8. doi: 10.1039/d6ra07548g (PMC13617610; doi:10.1039/d6ra07548g)
Supplement: Supplementary file 1 [file RA-016-D6RA07548G-s001.pdf]

Supplementary Information (SI) for RSC Advances.
This journal is © The Royal Society of Chemistry

Supplementary Information

Hybrid lipid-nanogels: nanolipogels for mRNA delivery

Ellen Oudkerk-Sodia,[a] Runxin Xu,[a] Renee Chu,[a] Rupali Dabas,[a] Sofia Patri,[a] Christopher Tidd,[a] Luka Blagojevic,[a] Rüdiger Woscholski*[a] and Nazila Kamaly*[a]

[a] *Department of Chemistry, Imperial College London, Molecular Sciences Research Hub, 80 Wood Lane, London W12 0BZ, United Kingdom.*

* Corresponding authors. E-mail: n.kamaly@imperial.ac.uk; r.woscholski@imperial.ac.uk

Contents

S1. Instrumentation

S2. Spectroscopic characterisation of phosphatidylbutenol

S3. Calibration curve for mRNA quantification

S4. Empty nanolipogels and synthesis optimisation

S5. Shelf-life of mRNA-loaded nanolipogels

S6. Supplementary TEM micrographs

S7. Fluorescent nanolipogel co-polymerisation scheme

S8. Statistical summary

List of supplementary figures

Fig. S1 ATR-FTIR spectrum and TLC of purified phosphatidylbutenol

Fig. S2 ATR-FTIR reference spectrum of DOPC

Fig. S3 High-resolution XPS C 1s spectra

Fig. S4 MALDI-TOF mass spectrum of phosphatidylbutenol

Fig. S5 RiboGreen calibration for mRNA quantification

Fig. S6 Size, PDI and zeta potential of empty AETC- and DMAEMA-NLGs

Fig. S7 Effect of polymerisation temperature on NLG quality

Fig. S8 Shelf-life of mRNA-DMAEMA-NLGs at −20 °C

Fig. S9 Additional TEM micrographs of mRNA-AETC-NLGs

Fig. S10 Additional TEM micrographs of mRNA-DMAEMA-NLGs

Fig. S11 Co-polymerisation scheme for fluorescent NLG synthesis

S1. Instrumentation

Dynamic light scattering and zeta potential. Hydrodynamic diameter, polydispersity index (PDI) and zeta potential were measured on a Malvern Zetasizer Nano ZS at 25 °C in disposable folded capillary cells. Each value reported represents the mean ± standard deviation of three independent measurements (each composed of 10–15 sub-runs).

Transmission electron microscopy. Negative-stain TEM was performed on a JEOL 2100Plus operating at 200 kV. Carbon-coated 200-mesh copper grids (TAAB Laboratories) were glow-discharged for 30 s, NLG dispersions (3 μL) were applied for 60 s, blotted, and stained with 2 % w/v uranyl acetate solution (3 μL, 30 s).

NMR. ^{1}H, ^{13}C and ^{31}P NMR spectra were acquired on a Bruker Avance 400 MHz spectrometer (^{1}H: 400 MHz, ^{13}C: 101 MHz, ^{31}P: 162 MHz) at 25 °C. Samples were dissolved in $CDCl_3$ (CIL); chemical shifts are reported in ppm relative to TMS (^{1}H, ^{13}C) or external 85 % H_3PO_4 (^{31}P).

FTIR. Attenuated total reflectance Fourier-transform infrared (ATR-FTIR) spectra were recorded on a PerkinElmer Spectrum 100 with a diamond ATR module, 4 cm⁻^{1} resolution, 16 co-added scans.

XPS. X-ray photoelectron spectra were recorded on a Thermo Scientific K-Alpha spectrometer using a monochromated Al Kα source (1486.6 eV). Survey scans (pass energy 200 eV) and high-resolution C 1s scans (pass energy 50 eV) were collected; spectra were referenced to adventitious C–C at 284.8 eV and analysed in CasaXPS.

MALDI-TOF MS. Mass spectra were acquired on a Waters Synapt G2-Si in positive-ion mode using α-cyano-4-hydroxycinnamic acid (CHCA) matrix (10 mg mL⁻^{1} in 70:30 MeCN:H_2O + 0.1 % TFA) deposited by the dried-droplet method.

Fluorescence and absorbance. mRNA quantification used the Quant-iT™ RiboGreen™ assay (Thermo Fisher) read on a BMG CLARIOstar Plus plate reader (λ$_{ex}$ 480 nm, λ$_{em}$ 525 nm), with confirmation on a NanoDrop One.

Confocal microscopy. Confocal images were acquired on a Leica inverted confocal microscope with 63× oil objective (HeLa, λ$_{ex}$ 405/633 nm; emission Hoechst 425–490 nm; Nile Blue 650–750 nm).

IncuCyte live-cell imaging. Live-cell green fluorescence and phase contrast were monitored on an IncuCyte S3 (Sartorius) at 37 °C, 5 % CO_2. Images were acquired every 2 h over 48 h; integrated green object intensity (GCU × μm^2 per image) was extracted using IncuCyte 2022B software.

S2. Spectroscopic characterisation of phosphatidylbutenol

Synthetic and analytical details for phosphatidylbutenol are given in Section 2.2 of the main text. The corresponding spectra are reproduced below.

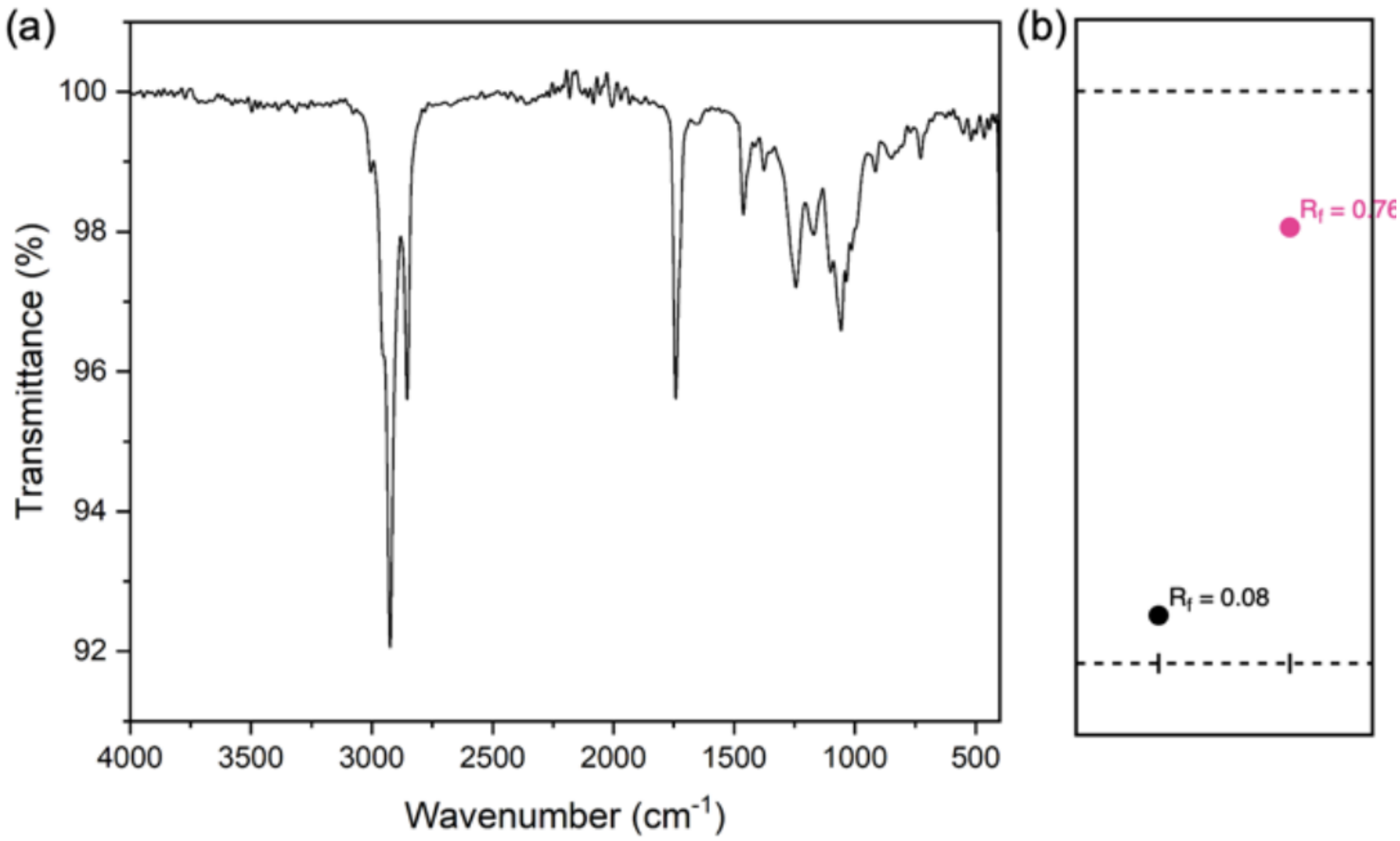

Fig. S1 (a) ATR-FTIR spectrum of purified phosphatidylbutenol; (b) retention factors (R_f = 0.71 and 0.08) of the species resolved by thin-layer chromatography of the reaction mixture. Key FTIR bands: 2924 / 2854 cm^{-1} (C–H stretch), 1741 cm^{-1} (C=O ester stretch), 1230 / 1063 cm^{-1} (P=O / P–O–C). The persistence of the alkene is corroborated by the C=C deformation near 1635 cm^{-1} (overlapping with the O–H bend of residual moisture).

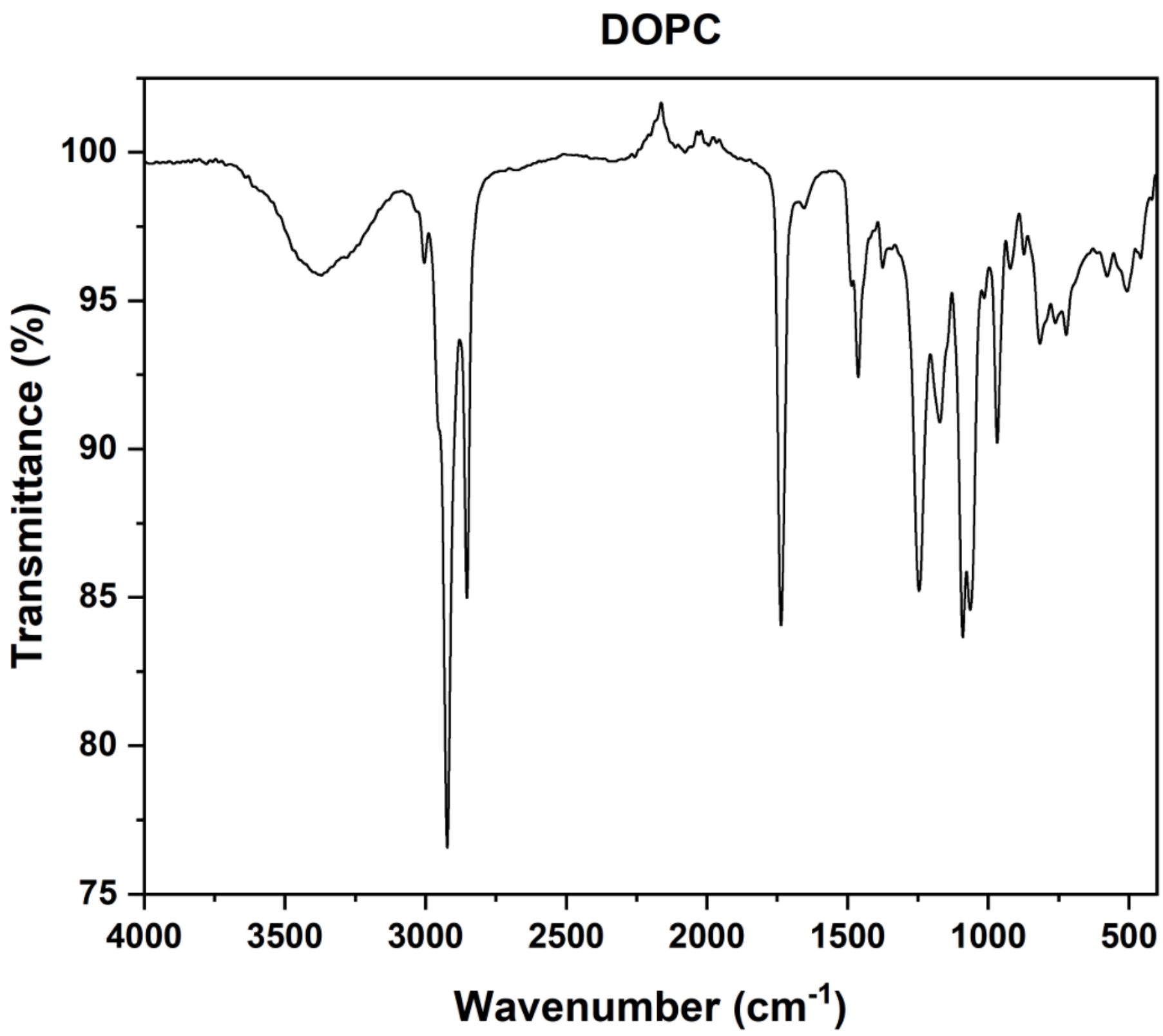

Fig. S2 Reference ATR-FTIR spectrum of the DOPC starting material (4000–400 cm⁻¹). Diagnostic phosphocholine bands at 1090 cm⁻¹ (P–O asym.) and 970 cm⁻¹ (N^+–CH_3 rocking) confirm the choline headgroup, which is absent after enzymatic conversion to phosphatidylbutenol (Fig. S1).

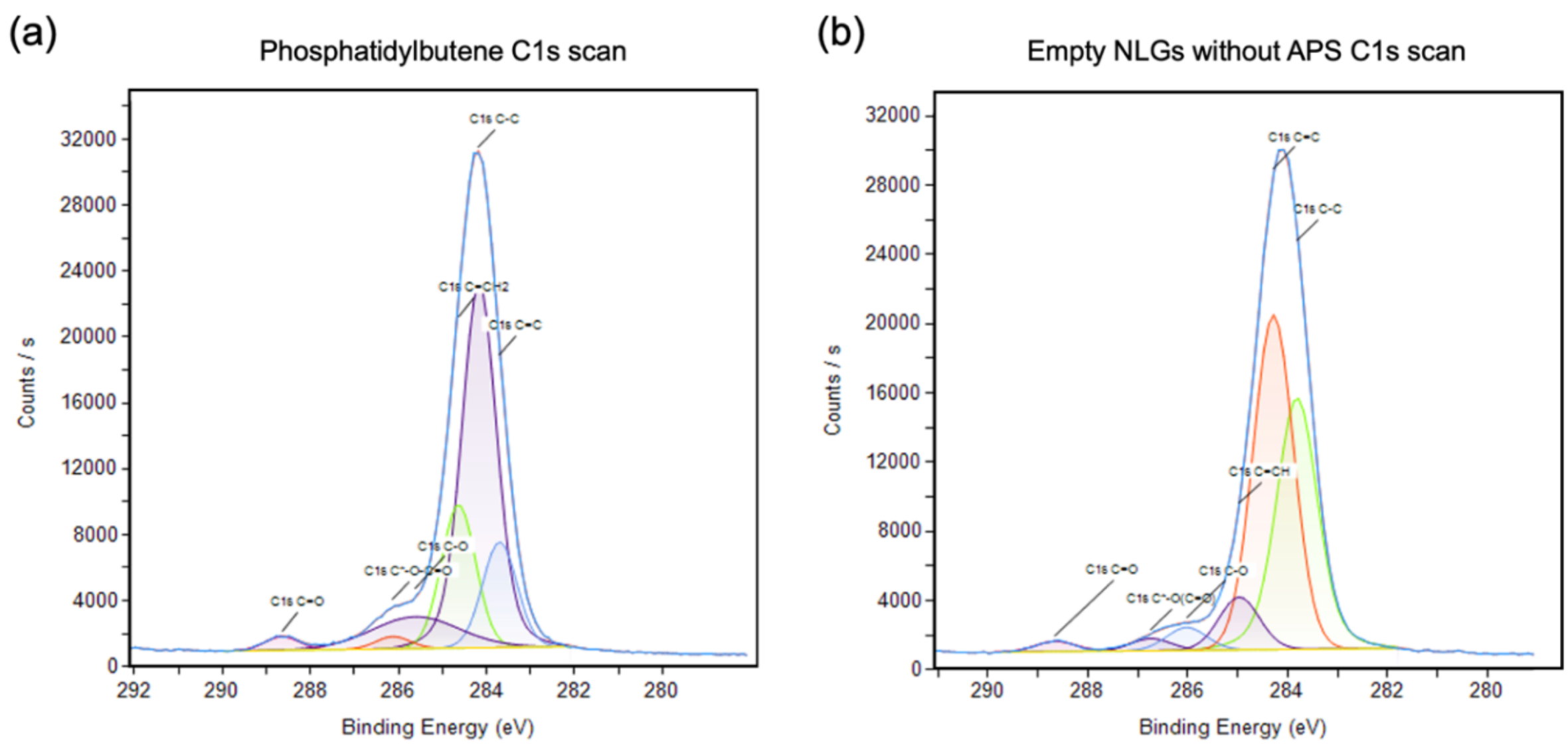

Fig. S3 High-resolution XPS C 1s spectra of (a) purified phosphatidylbutenol and (b) empty nanolipogels prepared without ammonium persulfate (i.e. no polymerisation). Peak fitting shows C–C (284.8 eV), C=C (285.1 eV), C–O (286.4 eV) and O=C–O (288.7 eV). Loss of the C=C component on incorporation into APS-polymerised NLGs (Section 3.2, main text) is consistent with consumption of the terminal alkene during free-radical co-polymerisation.

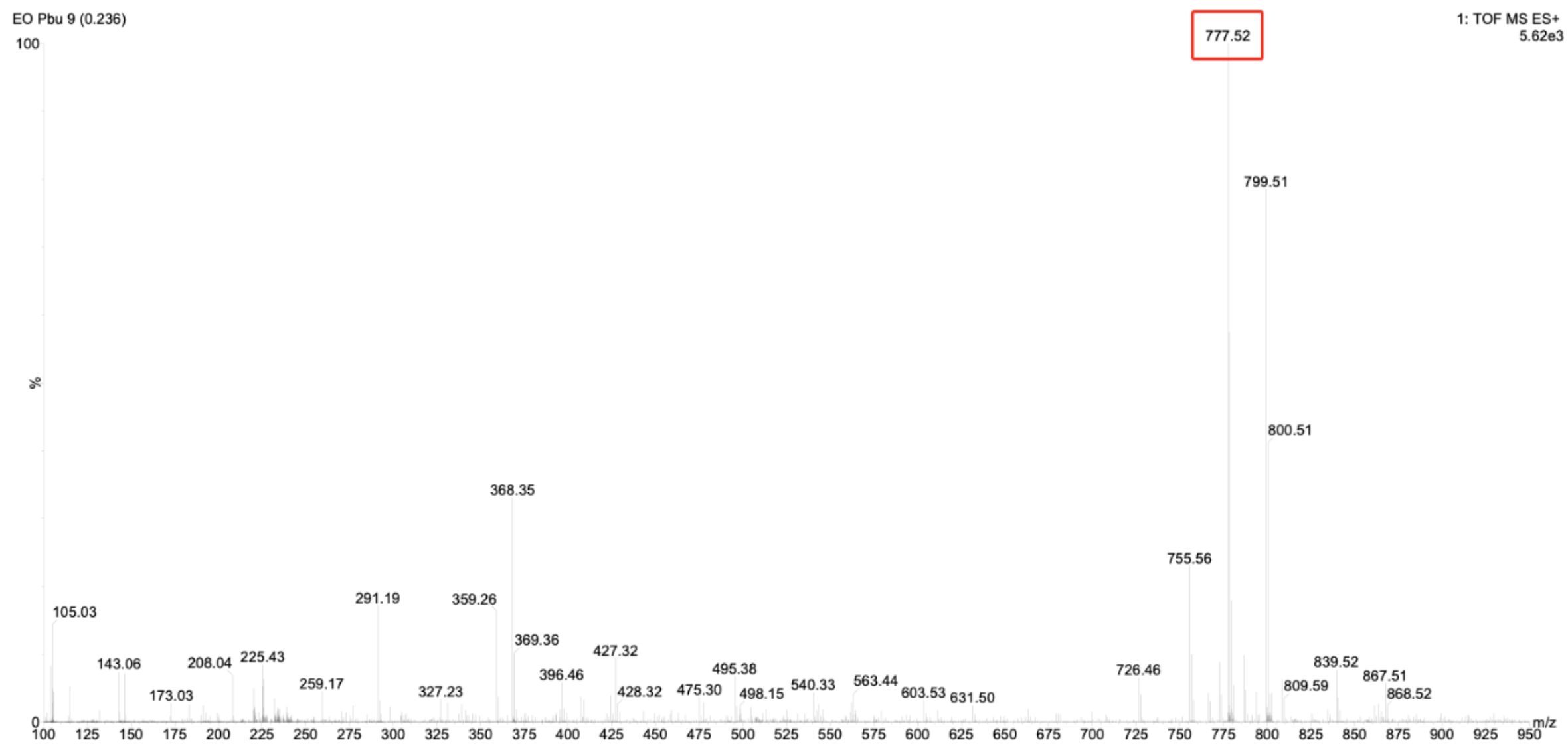

Fig. S4 MALDI-TOF mass spectrum of purified phosphatidylbutenol acquired in positive-ion mode with CHCA matrix. The principal ion at *m/z* 777.52 corresponds to $[M+Na]^+$ (calcd for $C_{43}H_{79}NaO_8P$ 777.55), with the accompanying *m/z* 799.51 ($[M+2Na–H]^+$) and *m/z* 755.56 ($[M+H]^+$).

S3. Calibration curve for mRNA quantification

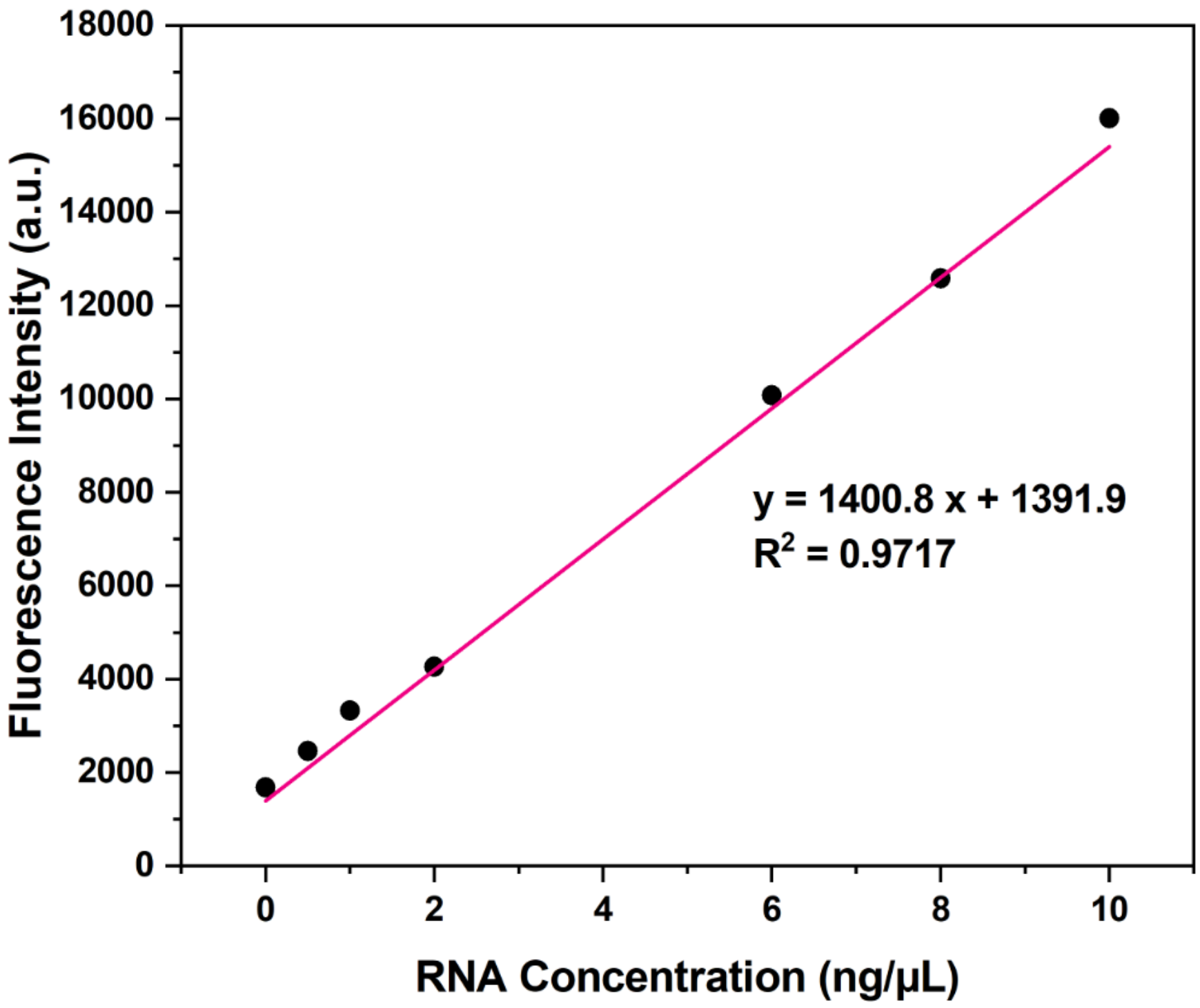

Fig. S5 RiboGreen mRNA fluorescence calibration. Fluorescence (λ$_{ex}$ 480 nm / λ$_{em}$ 525 nm) versus mRNA concentration: $y = 1400.8x + 1391.9$, $R^2 = 0.9717$, linear over 0–10 ng μL⁻^{1}. Encapsulation efficiencies reported in the main text (eqn (1)) were calculated against this curve.

S4. Empty nanolipogels and synthesis optimisation

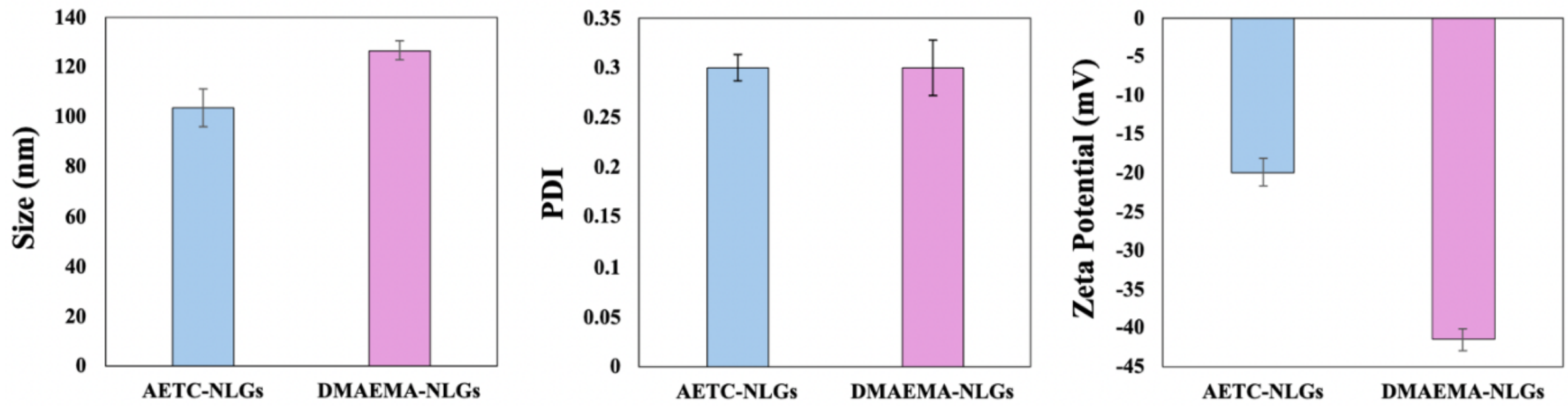

Fig. S6 Hydrodynamic diameter, PDI and zeta potential of empty AETC-NLGs and DMAEMA-NLGs (no cargo), measured by DLS (*n* = 3, ± s.d.). Empty AETC-NLGs: 103 ± 7 nm, PDI 0.30 ± 0.01, ζ −20 ± 1 mV. Empty DMAEMA-NLGs: 126 ± 4 nm, PDI 0.30 ± 0.03, ζ −42 ± 1 mV.

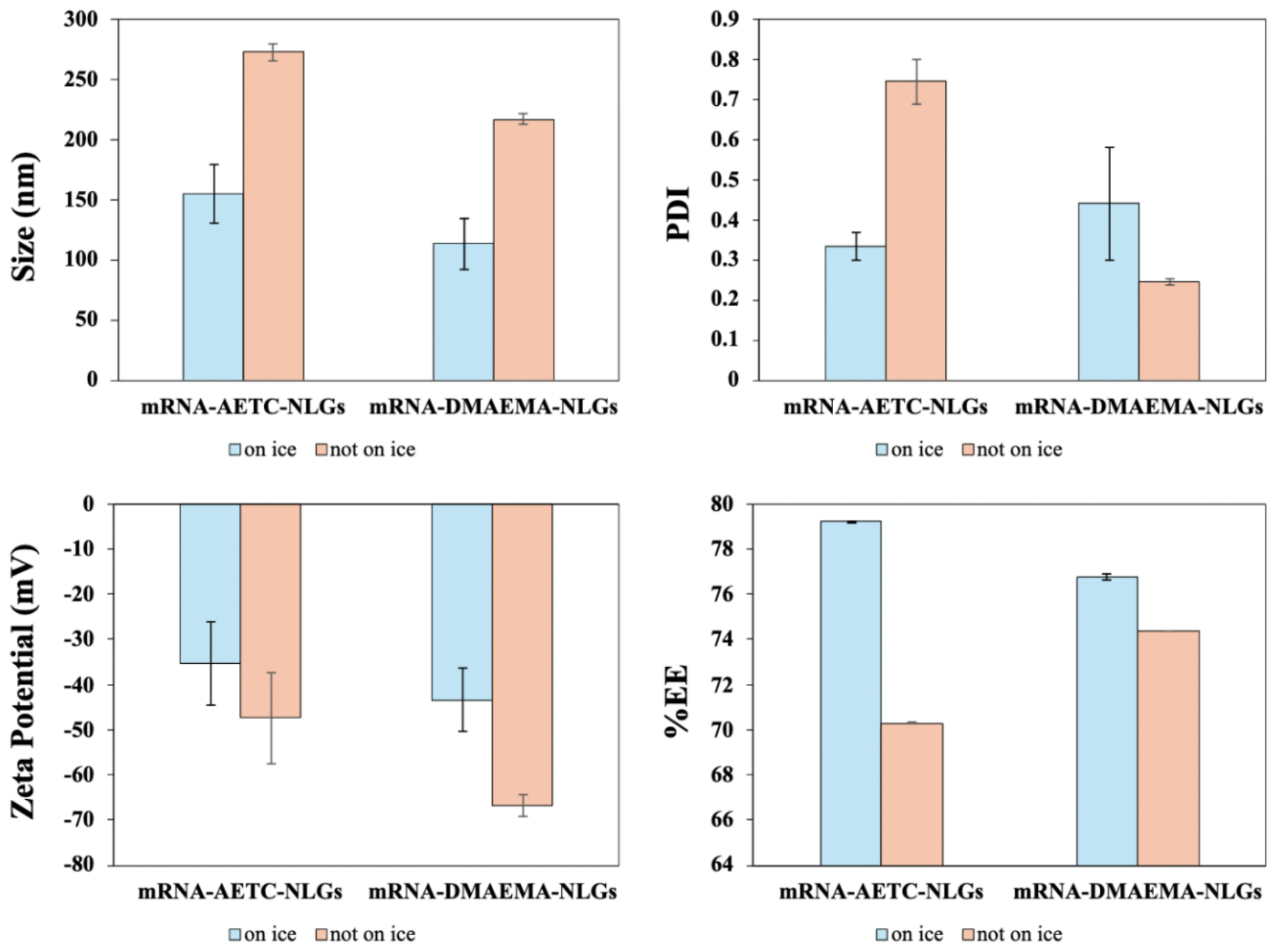

Fig. S7 Effect of polymerisation temperature on NLG quality. mRNA-AETC-NLGs and mRNA-DMAEMA-NLGs were synthesised under identical conditions either on ice (≈4 °C) or at room temperature (≈22 °C). Synthesis on ice consistently gave smaller particles, lower PDI, more negative zeta potential and higher mRNA encapsulation efficiency (%EE). All values mean ± s.d., *n* = 3.

S5. Shelf-life of mRNA-loaded nanolipogels

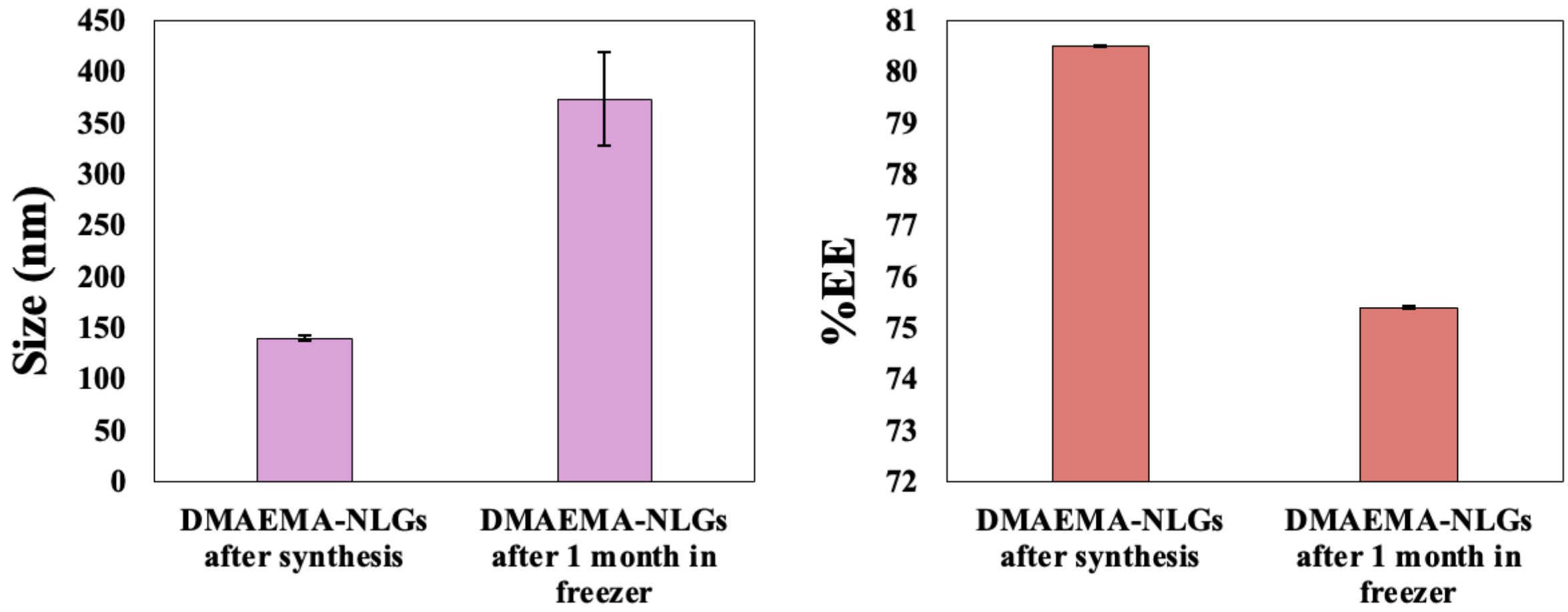

Fig. S8 Shelf-life of mRNA-DMAEMA-NLGs stored at −20 °C. Particle size and %EE were measured immediately after synthesis and after one month of storage. Hydrodynamic diameter increased from 140 ± 2 to 372 ± 45 nm; %EE decreased from 80.5 ± 0.05 to 75.4 ± 0.05 %.

S6. Supplementary TEM micrographs

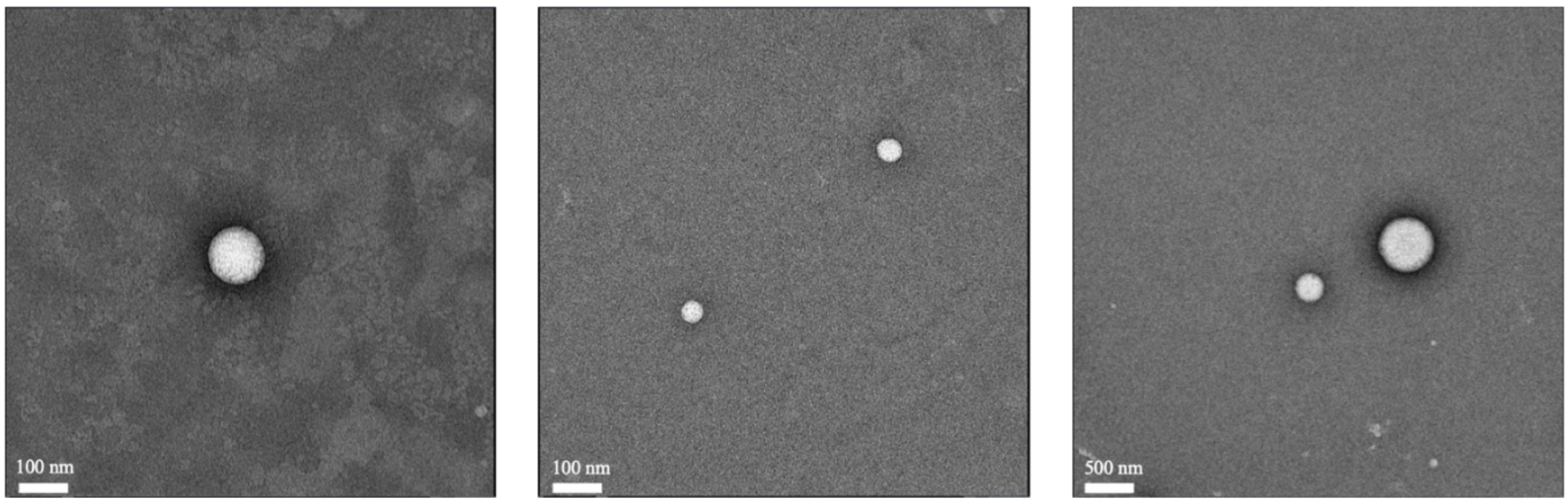

Fig. S9 Additional negative-stain TEM micrographs of mRNA-AETC-NLGs (scale bars: 100 nm, 100 nm, 500 nm). Particles are quasi-spherical, 50–150 nm in diameter, with smooth surfaces; aggregation is rare and consistent with the DLS PDI of ≈0.3.

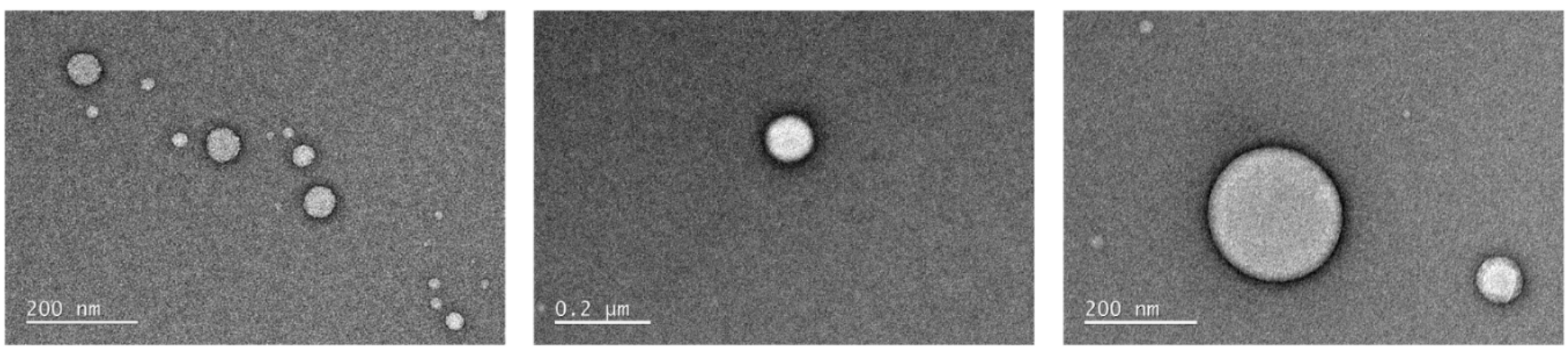

Fig. S10 Additional negative-stain TEM micrographs of mRNA-DMAEMA-NLGs (scale bars: 200 nm, 0.2 μm, 200 nm). Particles are larger and more polydisperse than AETC-NLGs, with a darker negatively-stained corona consistent with the mRNA-DMAEMA polyplex shell.

S7. Fluorescent nanolipogel co-polymerisation scheme

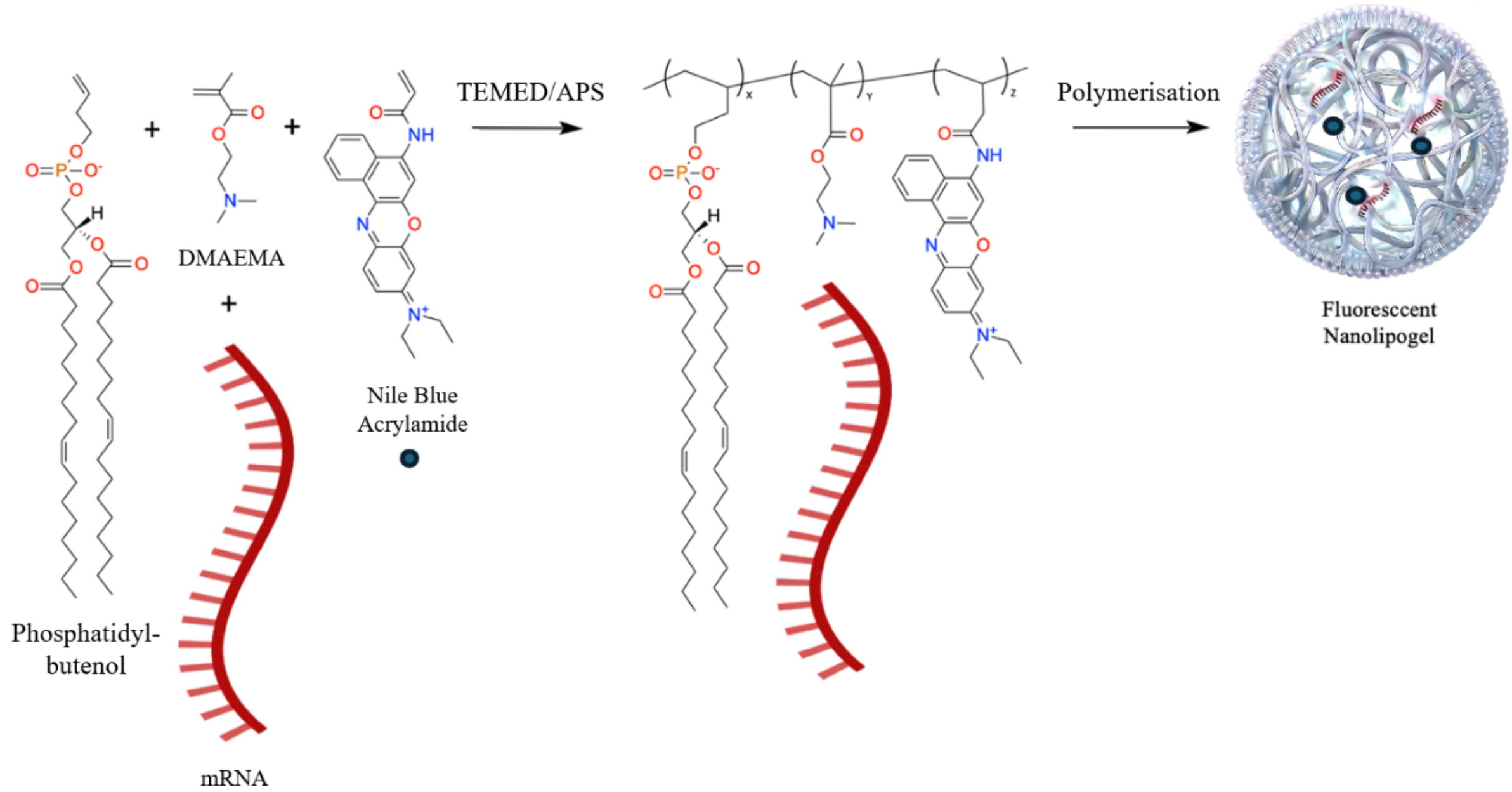

Fig. S11 Co-polymerisation scheme for fluorescent NLG synthesis (Section 2.6, main text). Phosphatidylbutenol, DMAEMA and Nile Blue acrylamide (5 mol % of total acrylates) are co-polymerised by TEMED/APS to give a covalently labelled fluorescent NLG that encapsulates mRNA without dye leaching. The relative sizes of the components are depicted schematically and are not to scale.

S8. Statistical summary

Statistical methods are described in Section 2.14 of the main text. The principal comparisons are summarised below.

CCK-8 viability (Fig. 4, main text). Cell viability of HeLa and bEnd.3 cells in the presence of mRNA-AETC-NLGs and mRNA-DMAEMA-NLGs at all tested concentrations (5–250 μg mL⁻^{1}) remained ≥85 % and was not significantly different from the untreated control by ANOVA ($p >$ 0.05).

IncuCyte transfection (Fig. 5c, main text). The transfection experiment was performed as a proof-of-concept study with one well per condition. At 24 h, total green-object integrated intensity was 1 116.9 × μm^2 per image for Lipofectamine 2000, 8 293.1 GCU × μm^2 per image for mRNA-AETC-NLGs, and 12 185.4 GCU × μm^2 per image for mRNA-DMAEMA-NLGs. As independent replicate wells were not included, these data are reported descriptively and no inferential statistical analysis was performed.
